# 

**Published:** 1874-11

**Authors:** 


### TABLE OF CONTENTS.

ORIGINAL COMMUNICATIONS.

Iridectomy as a Therapeutic Measure. By Swan M. Burnett, M.D., of Tenn. 625
Treatment of Retro-Flexions of the Uterus. By Ely Van DeWarker, M.D., of New York. - - - - - - 629
Incontinence of Urine in Children. By F. K. Bailey, M.D., of Tenn. - 636
A Case of Idiopathic Primary Abscess of the Orbit. By Swan M. Burnett, M.D., of Tennessee. - - - - - - 639
Green-Stick Fracture of the Clavicle, with Remarks. By Jno. N. Upshur, M.D., of Virginia. - - - - - - - 640
A Case and a Question. By Jno. W. Booth, M.D., of N. C. - - 641

REMARKS, GLEANINGS AND EXTRACTS.

The Therapeutic Uses of Aconite—643. On Syphilitic Insanity—644. Diagnosis of Heart Disease—646. The Communicability of Consumption—648. Influence of Anæsthetics upon the Sexual Impressions of Females—649. Incontinence of Urine in Children ; Life Sustained by Nutritive Enemata for a Period of Twenty-two Days—650. Cerebro-Spinal Meningitis—651. Change of Life; Castor Oil Vomited Up Forty Minutes after Being Injected Into the Rectum—652. Diagnosis of Stone—653. Phosphoric Acid in Urine in Cases of Disease of the Encephalon ; The Function of the Uvula ; Removal of a Needle from the Heart—Recovery—654. Maternal Impressions ; Local Treatment of Pulmonary Cavities by Injection through the Chest Walls ; Turpentine in Pyæmia—655. Excession a Portion of the Spleen—Recovery ; Case of Habitual Costiveness of Eight and a Half Months between Fecal Evacuations—656. A Difficulty in Fœtal Ausculation ; Sulphide of Calcium in Boils ; Lancing the Gums ; Bromide of Ammonium in Acute Articular Rheumatism ; Aspiration of Urine—657. Catarrh Snuff; Tracheotomy in an Infant—258. By T. Curtis Smith, M.D., of Ohio.

Physiological Action of Hydrocyanic Acid ; Hospital Observations Upon Negro Soldiers—658. Antidotes to Poisoning by Strychnine ; A Preparation for Treating Diarrhœa of Nursing Infants—659. Treatment of Inflamed Hemorrhoids in Parturient Women ; Neuralgia in Infants ; Local Action of Ipecacuanha—660. Recuscitation from Chloroform-Norcosis ; Phosphorus Pill ; Colorless Tincture of Iodine ; Purgative Mixture of Vienna—661. Danger of Intra-Uterine Injections ; Chloride of Potassium in Epilepsy ; Charcoal and Tar as a Surgical Dressing ; Treatment of Zona by Collodeon and Morphia—662. Nutritious Injections—a New Method of Nourishing Patients by the Anus ; Acute Ergot-Poisoning ; Iodide of Potassium in Acute and Chronic Bronchitis and Asthma—663. Is Labor Protracted by Early Spontaneous Rupture of the Membranes ; Chlorodyne ; Croton Chloral Hydrate in Intolerance of Light ; Chloral in Premature Labor—664. Notes of Hospital Practice, Charity Hospital, New York ; Treatment of Burns and Scalds ; Cannibal's Tastes—665. By Swan M. Burnett, M.D., of Tennessee.

Treatment of Putrescent Fever—666. Ergot in the Treatment of Nervous Diseases—667. The Radical Treatment of Hydrocele by Injection of Carbolic Acid—669. Chloride of Sodium in Dysentery ; Sleeplessness—570. Report Your Cases—671. Intra-Recto Abdominal Exploration ; Fistula in Ano ; A Large Appetite—672. Latest Modification of the Cod-Liver Oil Emulsion ; Hydrastin in Gonorrhœa—673. Ergot in Headache ; A New Sign of Pyelitis—674. Chloral as an Anæsthetic During Labor ; Waterproof Glue ; Purifying Water ; Lead in Syrups of Iodide of Iron—675. Galvano-Cautery ; The Efficiency of Enemata ; Sulpho-Carbolate of Zinc in Pruritis ; Toothache Anodyne ; A Strange Suggestion ; Formula for Summer Catarrh—676.

EDITORIAL AND MISCELLANEOUS.

Death of Dr. F. E. Anstie. - - - - - - - 677
A New Uterine Speculum ; The Medical Society of Virginia. - - 678
Bibliographical. - - - - - - - - - - - 679
List of Cash Payments to the Record. - - - - - - 684

